# Experimental and Analytical Study of under Water Pressure Wave Induced by the Implosion of a Bubble Generated by Focused Laser

**DOI:** 10.3390/s21144800

**Published:** 2021-07-14

**Authors:** Zhaofeng Han, Cyril Mauger, Thibaut Chaise, Thomas Elguedj, Michel Arrigoni, Mahmoud El Hajem, Nicolas Boisson

**Affiliations:** 1 Univ Lyon, INSA Lyon, CNRS, LaMCoS, UMR5259, 69621 Villeurbanne, France; zhaofeng.han@insa-lyon.fr (Z.H.); thomas.elguedj@insa-lyon.fr (T.E.); nicolas.boisson@insa-lyon.fr (N.B.); 2 Univ Lyon, INSA Lyon, CNRS, École Centrale de Lyon, Univ Claude Bernard Lyon 1, LMFA, UMR5509, 69621 Villeurbanne, France; cyril.mauger@insa-lyon.fr (C.M.); mahmoud.elhajem@insa-lyon.fr (M.E.H.); 3 ENSTA Bretagne, CNRS, IRDL, UMR6027, 29806 Brest, France; michel.arrigoni@ensta-bretagne.fr

**Keywords:** water cavitation peening, shot peening, PVDF sensor, Gilmore model, laser-induced bubble, optical cavitation, dynamic pressure

## Abstract

In various domains of material processing, such as surface cleaning and surface treatment, cavitation phenomenon may become an alternative to traditional methods if this phenomenon is well understood. Due to experimental and mathematical difficulties in theoretical models, it is still a challenge to accurately measure the physical mechanism of the fluid/structure interactions. In this study, we verified the feasibility of using polyvinylidene fluoride (PVDF) sensors to quantitatively measure the under-water pressure wave generated by the collapse of a single cavitation bubble. The electrical signal obtained by PVDF can be converted into pressure information only by using the sensor material parameters provided by the supplier. During the conversion process, only the capacitance of the acquisition chain needs to be additionally measured. At the same time, a high-speed video recording system was used to visualize the evolution of the cavitation bubble. The Gilmore analytical model and an associated wave propagation model were used to simulate the pressure peak of the first collapse of the cavitation bubble. This theoretical pressure was compared with the experimental results. The result showed that, for bubbles with a normalized standoff distance γ larger than 5, the PVDF sensor had the ability to quantitatively measure the pressure wave generated by a single cavitation bubble.

## 1. Introduction

Cavitation is a well-known but not yet fully understood phenomenon. This phenomenon occurs when the pressure drops sharply to the saturated vapor pressure [1]. The effects of cavitation are detrimental in many applications, such as marine technology and hydraulic facilities [2,3,4]. There are also some applications where cavitation is looked for and beneficial. In geophysics, electro-hydraulic devices, like sparkers, provoke a cavitation bubble and a shock wave that help in monitoring seismic activities [5]. In the automotive industry, fuel spray is produced by high pressure injectors.

The spray formation depends greatly on the flow characteristics at the nozzle orifice outlet, such as the turbulence energy and velocity distribution. The presence of cavitation within an orifice can also enhance the spray characteristics [6,7,8,9,10]. Some medical therapies take advantage of the mechanical impact of inertial cavitation, such as thromboltic therapy [11,12] and shock wave lithotripsy (SWL) [13,14,15,16,17,18]. Other therapies are based on the less destructive effects of stable cavitation bubbles, like sonoporation [19,20,21,22,23] or permeabilization of the blood–brain barrier [24,25].

In material processing, cavitation can be used for surface cleaning [26,27,28]. Shot peening can increase the life of mechanical parts by introducing compressive residual stresses at their surface. Traditionally, shot peening is performed by grain blasting. Two decades ago, shot peening induced by cavitation was proposed and developed by Soyama et al. [29]. This technique also improves the mechanical performances of the treated material. This process was named Water Cavitation Peening (WCP). It involves submerging the surface of the part in water and spraying high-speed water on the treated surface.

Due to the huge shear force between the water jet and liquid environment, a strong vorticity appears at the nozzle outlet. As stated by Soyama [30], the vortices play an important role in the cavitation process. Through experiments, it has been proven that both ring vortices, single helical vortices and double helical vortices exist in the cavitation flow [31,32,33]. The pressure inside the vortex can drop below the saturated vapor pressure of water and will, then, vaporize the fluid. Then, cavitation bubble clouds/pockets will be generated in the water and carried by the flow.

Experimental studies have been performed to measure the induced effect of the cavitating jet. Klumppa et al. [34] proved that for AISI4140 cavitation peening can provide the same level of compressive residual stress as shot peening, but with a higher penetration depth. Soyama et al. [35] showed the application of cavitation peening in soft materials (e.g., aluminium alloys) with a good ability to improve its fatigue strength without any mass loss. Laser cavitation peening was also investigated by Soyama [36] and could improve the fatigue strength of stainless-steel welds, and he used a polyvinylidene fluoride (PVDF) sensor and a conventional submerged shock wave sensor to qualitatively evaluate the impact forces induced by laser ablation and laser cavitation collapse.

The conventional submerged shock wave sensors, like crystal and quartz base sensors, have a lower frequency band (some kHz); therefore, the PVDF sensor is more suitable for impact measurement. In a recent study by Reuter and Ohl [37], a 5 million frame per second high-speed imaging system with femtosecond illumination technology proved that the collapse of bubble close to a rigid boundary may transform into a micrometer-sized, supersonic needle jet. This needle jet will produce a higher pressure than classic micro-jets. This effect might be of first order in cavitation peening or cavitation erosion.

To further study the process of cavitation peening, it is necessary to predict the mechanical loading during this process. Models for the prediction of the cavitation peening process have recently been developed and partly based on the single bubble dynamics [38]. This work combined the pressure wave generated by a single bubble with the volume fraction distribution in the cavitation stream obtained through Computational Fluid Dynamics (CFD) modeling in order to predict the pressure generated by the cavitating jet. The enhancement of such a predictive model requires precise experimental data. Therefore, the observation of single bubble dynamics and accurate measurements of the pressure waves in experiments remain critical.

In the past 100 years, the research on the dynamics of a single cavitation bubble has never stopped. Some focused on observing the dynamics of a single bubble generated in water, the phenomenon of single-bubble sonoluminesence and shock wave generated by bubble collapse [39,40,41,42,43,44,45], some focused on aspherical bubble generated near a solid boundary or elastic boundary [46,47,48] and some also studied bubble dynamics in viscoelastic materials [49,50].

Under the condition of achieving a simple and repeatable procedure, there are three main methods to induce independent bubbles, using underwater discharge, ultrasonic waves and focused laser pulses. Wang and Chen [43] discharged a capacitor composed of tungsten electrodes to generate a bubble at different distances from a solid boundary. At the same time, they used a piezoelectric polymer made of polyvinylidene fluoride (PVDF) to measure the pressure wave generated by cavitation bubble collapse, and used the signal wave as a basis to explain the process of the bubble collapse in different ranges of distance from the solid boundary.

Nevertheless, in their study, the PVDF sensor required a calibration (pendulum-type ball impact technique) to obtain a quantitative pressure measurement. This calibration process consists of determining the relation between the produced electrical charges and the pressure that produces the piezoelectric effect. Another method for producing cavitation bubbles consists of the use of acoustic waves, such as ultrasonics [51], but it is difficult to control the number and location of bubbles. In the laboratory environment, using a focused laser pulse to produce size-controllable bubble is probably the most widely used method [42,52,53,54].

Lauterborn [52] used laser-induced bubbles in water and observed the dynamics of bubble collapse in detail with a high-speed camera (optical cavitation). Tomita and Shima [55] explained the entire process and method of laser-induced bubble in water with more details, and also introduced the methodology of controlling the laser beam and its energy to achieve the controllability of generating bubbles.

In all these studies, a rapid imaging system with high-speed camera made it possible to observe a single bubble resulting from a high energy transmission into water. However, all of them pointed out the fact that pressure remains a challenge to measure because of the briefness of the pulse, in the order of nanoseconds, high intensity of the pressure, which can reach the level of GPa [56,57], and measurement surfaces of the sensors that are too large (some mm^2^) in short range with respect to the shock wave curvature. In order to determine the pressure wave generated by the bubble collapse, a Fiber Optic Probe Hydrophone (FOPH) has been used by Wang et al. [58].

Sinibaldi et al. [59] also used FOPH to determine the pressure field of laser-induced bubbles, in order to study the effects of laser focusing angle and laser energy on bubble generation. From these applications, FOPH seems to be a good equipment to measure the pressure wave. The principle of FOPH is to record changes in the refractive index of the liquid caused by pressure waves, its accuracy varies with different aspect, like the temperature of water, the possible presence of impurities in water and the distance between the bubble center and sensor. PVDF film sensors are another technology that has the ability to measure the pressure wave directly.

Bauer [60] showed successful applications of PVDF sensors in different experimental scenarios, different fields of applications with a capacity of pressure measurement in a range from kPa to GPa and nanosecond resolution [61]. Bauer and Lichterberg [62] used PVDF to realize the measurement of high dynamic pressure in Hopkinson bar and for low-impedance materials [63].

Toda and Thompson [64] created a vibration sensor by combining a silicone rubber contact head with a curved PVDF film. Furthermore, compared to piezoelectric ceramics, PVDF has an acoustic impedance close to water for higher sensitivity. Under the same shock, PVDF tends to provide higher output voltage. At the same time, thanks to its flexible PVDF film, it can be more easily attached to surfaces with various shapes. In the case, considered in this work, the PVDF film sensors are, therefore, a suitable choice.

There were many studies conducted to predict the dynamics of spherical bubble collapse and the resulting pressure wave. The first model was proposed by Rayleigh [65] to describe the dynamics of an isolated spherical bubble in an incompressible medium. In fact, the hypothesis of incompressibility of the liquid did not allow taking the shock waves into account. Gilmore [66] proposed a more accurate model using the Kirkwood–Bethe approximation. This assumed that wave propagation in the liquid occurs at sonic velocity.

Nevertheless, this approximation becomes inaccurate for high Mach numbers [67]. Gilmore’s model has the particularity to account for the growth and collapse of a spherical bubble. At the same time, it considers the second-order compressibility terms, which allows for a better explanation of the fluid compressibility effect. Keller and Miksis [68] provided a model based on a constant speed of sound in the liquid and the compressibility is also considered.

The purpose of the present work is to verify the feasibility of quantitative measurements of a cavitation bubble-induced pressure wave using PVDF sensor without preliminary calibration. It is worth explaining that the sensor itself is not calibrated meaning that the data provided by the manufacturer are sufficient to obtain a quantitative measure of the pressure without specific calibration. The value of the piezoelectric coefficient $d_{33}$ that results from the manufacturing process of the sensor was validated previously with experiments using notably a shock tube [60,69].

The paper is organized as follows. The apparatus set-up to generate the cavitation bubbles, record their dynamic and acquire the pressure variation in water is presented in Section 2. Section 3 provides examples of experimental data obtained. The Gilmore analytical model, and the associated pressure wave propagation model as well as the process of obtaining initial conditions are summarized in Section 4. The effect of the curvature of the spherical pressure wave and the effect of shock wave propagation in different mediums are explained in detail in Section 5. The comparison between the experimental data and the analytical model is presented in Section 6. Finally, our conclusions and outlooks are given.

## 2. Experimental Set Up

**Figure 1:**
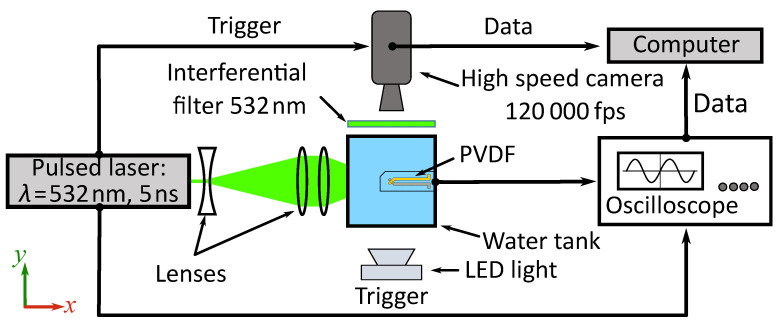
Schematics of the experimental set-up.

The experimental setup needs to ensure three functions. It has, first, to generate a cavitation bubble with controllable size. Secondly, this bubble has to be observed at high frequency in order to catch its dynamics. Finally, the under water pressure variations associated with the bubble dynamics, including its implosion, have to be measured using a PVDF sensor. An experimental set-up was developed to achieve these three functions. Figure 1 shows the experimental setup used to generate bubbles and observe the bubble dynamics in synchronization with under water pressure measurement.

For convenience and reproducible aspects, the laser-induced bubble method was chosen to generate the bubbles. A Q-switched Nd:YAG laser, in Figure 1 (New Wave Research Solo III, λ=532 mm), the pulse length is 5 ns and pulse energies were around 10 mJ. The laser beam was focalized into a 6 cm cubic side water tank (made of glass) to produce bubbles inside. The laser beam was widened by passing through a diverging lens (f=−25 mm) and then collimated by a convergent lens (f=250 mm).

Finally, the beam was focused by an aspherical lens (f=40 mm) in micro-filtered and demineralized water. The focused energy must be sufficient to reach the ionization temperature of water and generate a plasma, which will rise to a vapor bubble. According to the experience gained in the frame of laser matter interaction [70,71], for nanosecond pulses, at higher power densities (>${GW\;cm}^{-2}$), optic breakdown occurs. In the studied case, the laser energy is about 10 mJ, which is sufficient to produce optic breakdown in the focus. By tuning the laser energy, it is possible to control the bubble size generated in the water tank.

The second function of this experiment platform is to capture the bubble dynamics. The main equipment is a CMOS camera (Vision Research V12.0) equipped with a 12× objective lens (Navitar). A frame size of 128×256 pixels and an acquisition frequency of 120 kfps were used for the recording. Backlight illumination was assured by a continuous light-emitting diode (3 W LED).

A band reject filter (Notch filter 532±12 nm) protected the camera sensor from laser lighting. With an opening time of 300 ns, the image was perfectly frozen. The magnification of the image was 13.7 μmpx^−1^. The recording of the high-speed camera and the pressure acquisition by the PVDF sensor presented below are synchronized with the Q-switch of the laser.

We observed that the widths of the shock waves in the range of 10 ns–100 ns and that the maximum pressure at the center of the bubble were of several GPa [44,54]. Thus, a sensor with a large bandwidth and the ability of fast acquisition is needed. For such high frequencies and pressure amplitudes, the Bauer pressure shock sensor was borrowed from shock physics [61]. This technique is based on the use of a piezoelectric polymer made of a PVDF stretched film. Technical specifications of the Bauer shock sensor utilized in the presented work are given in Table 1.

**Figure 2:**
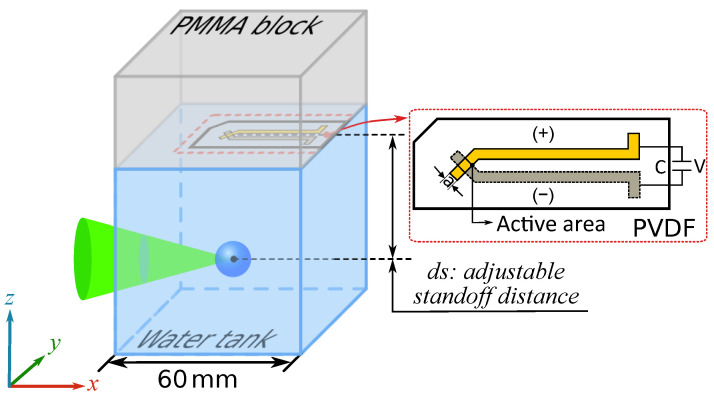
Schematic of the relative position of bubble in the water tank, the installation position of the PVDF sensor and the structure of the PVDF sensor.

The acquisition chain with PVDF sensor, oscilloscope and computer is presented in Figure 1. The position of the PVDF sensor in the experimental set-up and the structure of the PVDF sensor are shown in Figure 2. Poly (methyl methacrylate) (PMMA) was chosen as backing material because of the similarity of its impedance to PVDF. The PVDF sensor was pasted on the center of a 40 mm thickness PMMA block. The PMMA block with the PVDF sensor was placed directly on top of the water tank, and water was manually added between the PMMA and water tank to ensure that there was no air at the interface. This allowed us to minimize the influence of the cavity between the PVDF sensor and the water surface and to limit the wave reflections.

To observe the signal of the shock wave, the PVDF sensor was connected to a high-performance oscilloscope (Keysight InfinitiVision DSOX3054T) in voltage mode. The characteristic time RC is estimated by multiplying the resistance (1 MΩ) and the total capacitance of the acquisition chain ( 26.5 pF); thus, the RC time was about 27 μs longer than the observed pulse duration. One can refer to Arrigoni and Bauer [61] for further details. The oscilloscope could catch the data with a bandwidth of 500 M and a sampling rate of 625 MSas^−1^ in high impedance mode (1 MΩ). The time resolution could reach 1.6 ns.

The relative position of the bubble and the center of the sensor is of importance for the analysis of the results. When the sensor is off axis with respect to the bubble center, the shock front curvature does not remain frontal to the sensor’s active area. Like Figure 2, in the horizontal (x,y) plane, it is necessary to ensure that the center of the bubble coincides with the center of the PVDF active area. In the vertical direction (*z*-axis), the influence of the standoff distance $d_{s}$ between the bubble center and the PVDF sensor is an important parameter that will be studied in this work. In order to realize the variable standoff distance $d_{s}$, the water tank can be moved in the *z* direction with a 0.1 mm precision.

## 3. Experimental Result

### 3.1. Bubble Dynamics

**Figure 3:**
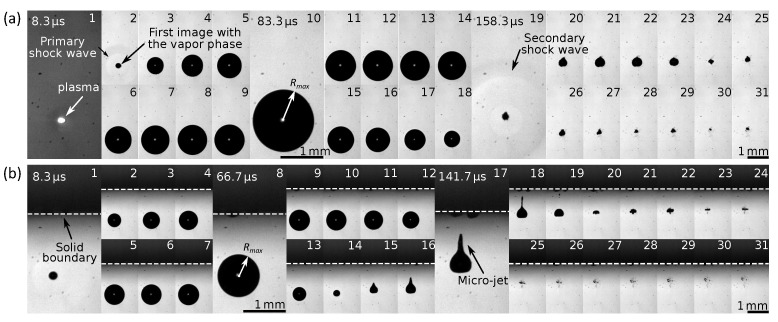
(**a**) Dynamics of a cavitation bubble far from a solid boundary. (**b**) Dynamics of a cavitation bubble in the close presence of a solid boundary. The time between each image is constant, and the frame rate is 120 kfps.

Figure 3 shows two image sequences taken at 120 kfps. These sequences make it possible to follow and compare the evolution of a bubble created by a focused laser at different standoff distances from the PVDF sensor. When the laser energy is concentrated in a very small volume of water (r<100μm), the water is heated to ionization temperature [54]. After that, this part of the heated liquid is converted to a thermal plasma (Figure 3a-1). The plasma rapidly expands and compresses the surrounding liquid. A spherical pressure wave is generated in this process, and then the surrounding liquid is accelerated radially.

**Figure 4:**
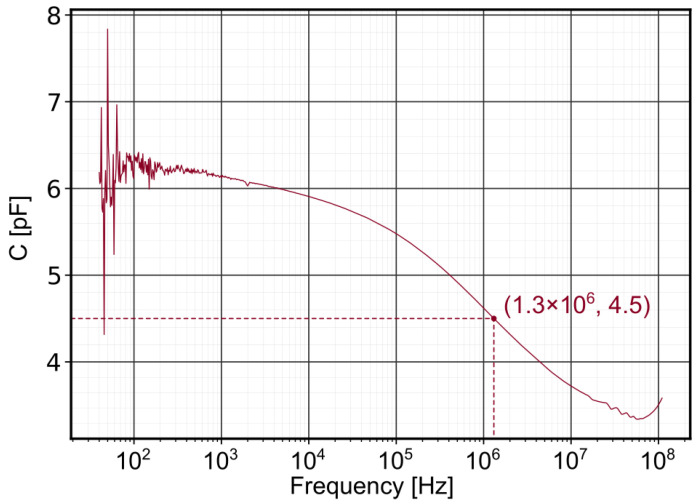
Capacitance of the PVDF sensor (1 mm^2^) with an oscillator level of 500 mV over a range of 40 Hz to 500 MHz.

**Figure 5:**
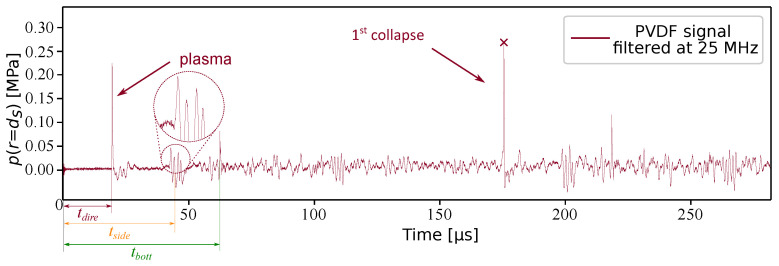
Quantitative measurement by a PVDF sensor for γ=36.4. The red cross is the maximum values of the pressure wave generated by the first collapse of the bubble.

**Figure 6:**
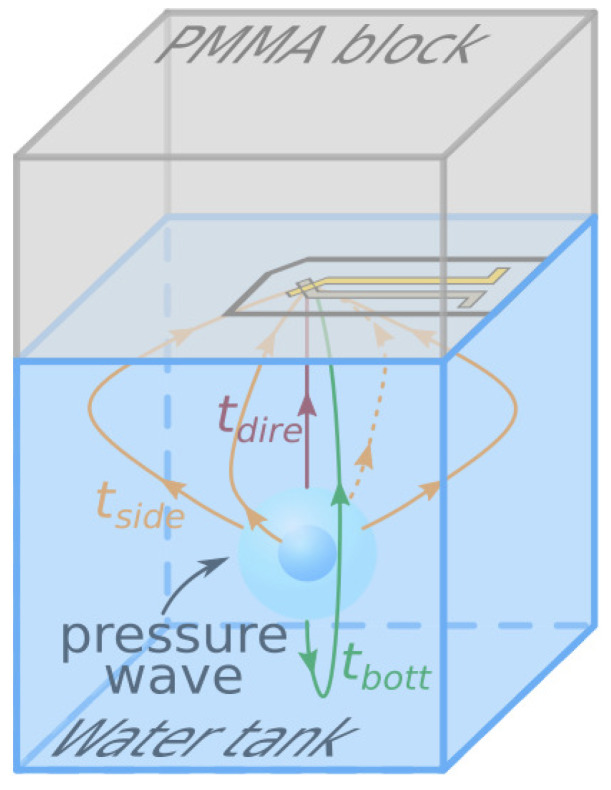
Schematic diagram of direct propagation of pressure wave and multiple reflections. The red path is the direct propagation, the orange paths are sidewall reflections and the green path is the bottom reflection, the time required for the propagation are, respectively, $t_{\mathrm{dire}}$, $t_{\mathrm{side}}$ and $t_{\mathrm{bott}}$.

**Figure 7:**
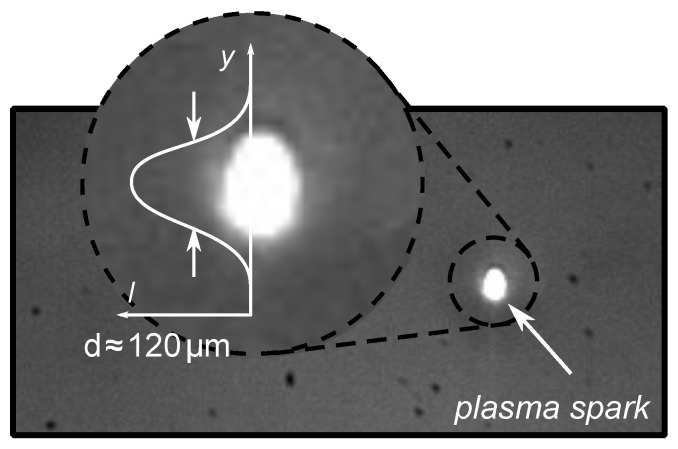
Example of the measurement of the plasma size d≈ 120 μm.

**Figure 8:**
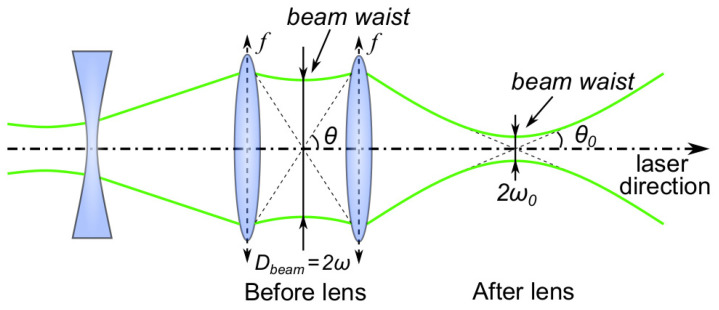
Schematic diagram of the laser path through the lens set.

**Figure 9:**
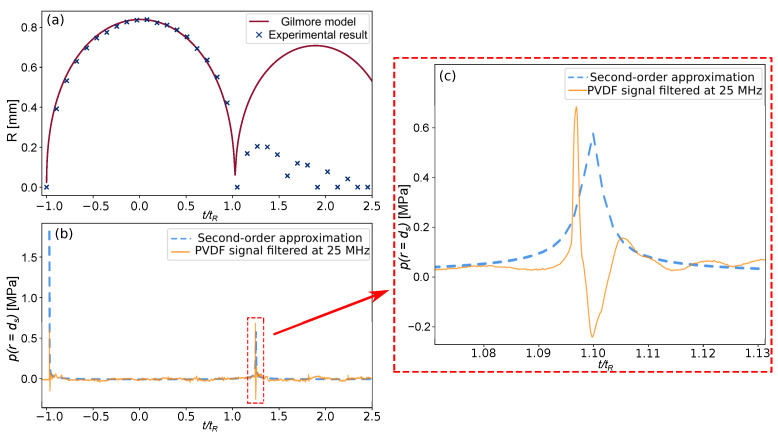
(**a**) Evolution of the bubble radius: comparison between experimental data and the Gilmore model. (**b**) Comparison of the pressure evolution with γ=10.1 from the PVDF sensor and wave propagation model. (**c**) Zoom of the peak pressure caused by the bubble collapse.

**Figure 10:**
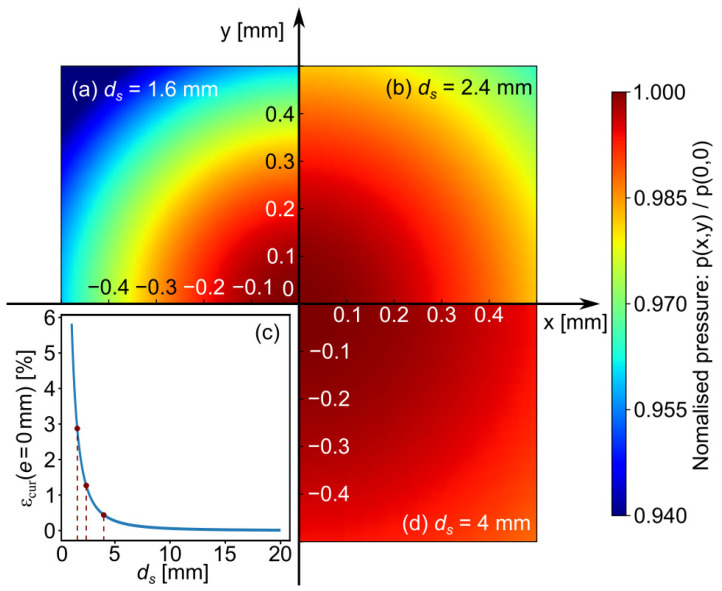
Examples of theoretical disparity caught by the PVDF sensor for different standoff distances $d_{s}$, with a constant $R_{max}$ (0.8 mm). All the pressure values were normalized by the maximum pressure at the center of PVDF sensor. (**a**) $d_{s}$ = 1.6 mm, (**b**) $d_{s}$ = 2.4 mm, (**d**) $d_{s}$ = 4 mm. (**c**) Estimation of the error $\varepsilon_{\mathrm{cur}}$ for different standoff distances $d_{s}$.

Finally a bubble is generated (Figure 3a-2 and b-1). As the plasma pressure is isotropic, the bubble expands almost spherically. The bubble grows to a critical size $R_{\mathrm{equilibrium}}$ where the pressure inside the bubble is equal to that of the outside. At this moment, due to the inertial forces, the bubble has expanded to its maximum size $R_{\max}$ (Figure 3a-10 and b-8) where the pressure inside the bubble is smaller than the pressure in the water environment, and a contraction motion starts to occur.

Then, the bubble enters in a collapse phase. Researchers observed that, when the bubble reaches the maximum radius, the thermodynamic equilibrium is re-established, and the bubble dynamics are no longer affected by the initial thermal plasma [72]. In addition, research demonstrated that the characteristic times associated with the propagation of heat in water were considerably longer than those of pressure waves [73]. Therefore, the behavior of the bubble is, thus, similar to that of a cavitation bubble in the collapse phase. At the end of the contraction phase, because of the inertial effects, the size of the bubble will continue to decrease, while the pressure inside the bubble continues to increase, and it will cross the equilibrium size $R_{\mathrm{equilibrium}}$ once again.

This process continues until the bubble shrinks to a minimum volume. At this moment, the pressure inside the bubble is greater than the ambient pressure in the water. This sudden contraction generates a highly increasing pressure rate that results in a secondary shock wave visible in Figure 3a-19. After this, the process of rebounds and collapse repeats until the non-condensable gases remaining in the bubble dissolve. This process is similar to that observed during underwater detonation of high explosives [74]. During these phases of implosion, under certain conditions, it is possible to observe the fragmentation of the bubble into smaller bubbles. This was observed by Tomita and Shima [55], but this situation was not considered in this study.

When away from a solid boundary, the bubbles collapse in a quasi-spherical manner. A perfectly spherical collapse can only be achieved under special conditions where no hydrostatic pressure gradient influences the bubble [54]. In the case of a solid boundary close to the bubble in Figure 3b, the bubble will collapse in an asymmetrical manner. This can create a very fast liquid micro-jet directed toward the solid boundary. For the process of cavitation peening, the primary shock wave generated by a perfect spherical collapse had a higher pressure level than an aspherical collapse. This conclusion has been obtained both experimentally by Soyama [75] and computationally analysis by Sonde et al. [38].

The normalized standoff distance to the solid boundary γ is defined as: $\gamma =d_{s}/R_{\max}$, where $R_{\max}$ is the maximum radius and $d_{s}$ is the standoff distance between the center of the bubble and the solid boundary. The pressure fluctuation generated by the bubble dynamics can be measured by the PVDF sensor for various γ.

### 3.2. PVDF Signal Processing

The rapid expansion and collapse of bubbles is accompanied by the emission and propagation of pressure waves in the water. When the pressure wave propagates and impacts the surface of the PVDF sensor, the active area of the sensor undergoes a slight deformation. Due to the piezoelectric ability of the PVDF, the two poles of the PVDF sensor will produce a voltage change under the action of the pressure.

The voltage evolution will be detected and recorded by the oscilloscope introduced in Section 2. When the shock wave hits the sensor, a shear wave is also generated. The $d_{31}$ and $d_{32}$ coefficients are equal to 5.9±3% pCN^−1^, four times lower than $d_{33}$; therefore, this effect is usually considered to have a negligible effect on the signal generated by the sensor.

This voltage signal can also be converted into pressure intensity based on the PVDF sensor and the acquisition chain characteristics. The sensor used in the experiment was only characterized in the direction perpendicular to its surface, which has no influence on the results, as no shear loading was applied to the surface. The following relations have been used:

(1) Q=CU,

(2) $$p=\frac{F}{S},$$

with *Q* as the charge value on both sides of the PVDF sensor, *C* as the total capacitance of the acquisition chain and *U* as the voltage value measured by the oscilloscope. The pressure *p* affecting the sensor active surface *S* is linked to the resulting force *F* applied to the surface. According to the PVDF characteristics, the charge and the force are linked by:

(3) $$Q=d_{33}F,$$

where $d_{33}$ is the piezoelectric coefficient mentioned in Table 1. By combining Equation (1) to Equation (3) the relation between charge and pressure becomes:

(4) $$p=\frac{CU}{d_{33}S}.$$

From Equation (4), the voltage signal is linked to the pressure intensity using the sensor parameters provided by the supplier and presented in Table 1. Only the capacitance of the acquisition chain needs to be additionally measured, other than that, no calibration is required. Although the supplier gives a capacitance reference value for the sensor, the capacitance is frequency dependent. The capacitance of the PVDF has, therefore, been measured over a large frequency range with a capacitance meter (Agilent 4294A).

The capacitance of the sensor was scanned under an oscillatory sollicitation of 500 mV over a frequency range of 40 Hz to 500 MHz. In Figure 4, the change of the capacitance of PVDF in the full frequency domain can be seen. From the data provided by the oscilloscope, the average frequency value under impact is 1.3 MHz. In this case, a more accurate capacitance of the PVDF sensor is taken as 4.5 pF. The total capacitance of the acquisition chain is, finally, $C=C_{\mathrm{osci}}+C_{\mathrm{pvdf}}=26.5\;pF$, with $C_{\mathrm{osci}}=22\;pF$.

The PVDF sensors are, here, in a range of linear response for pressures below 100 MPa [60]. Note that these PVDF sensors have been used for higher pressure values (up to tens of GPa). Above the linear domain, the correlation between voltage and pressure is still possible using the data provided by Mostovykh and Arrigoni [76].

#### Identification the PVDF Signal

Figure 5 shows the signal recorded by the oscilloscope for the case of γ=36.4 and a standoff distance $d_{s}$ of 29 mm, which is triggered at t=0. The red curve corresponds to the pressure signal converted from the voltage produced by the PVDF sensor using Equation (4). In this work, a Butterwort filter was used with a cutoff frequency of 25 MHz. The data was processed in both the forward and reverse direction to maintain zero-phase. The signal before and after filtering are compared in Appendix B, showing the negligible influence of the filtering on the recorded results.

Knowing the position of the bubble center, the dimensions of the tank, as well as the moment when the plasma is generated, it is possible to identify the peaks corresponding to the different types of wave reflections by calculating the time of arrival of the corresponding waves. These times are illustrated in Figure 6. The first pressure wave matches with the plasma generation. It is reflected on the walls of the glass tank before the second pressure wave, which is generated by the first pulsation of the bubble.

The time of arrival of the pressure wave generated by the plasma $t_{\mathrm{dire}}$ (the red path in Figure 6) can be easily calculated: $t_{\mathrm{dire}}\approx 19.5\mu s$. This time is in line with the reading time between the trigger moment and the first peak. The second peak is, in fact, a group of peaks, which is zoomed in Figure 5. It can be seen in the enlarged view that the group of peaks is composed of four waves reflected by the four side walls (the orange track in Figure 6).

Since these four peaks arrive on the PVDF sensor at slightly different times, it can be inferred that the position of the bubble is not exactly at the center of the cube, but with a slight deviation. The time of arrival of the third peak is related to the pressure wave reflected on the bottom wall (green track in Figure 6). This peaks sequence is repeated for each bubble collapse that follows. Nevertheless, since the collapse energy is less and less significant after the first pulsation, this can hardly be seen on the signal. The red cross indicates the maximum value of the pressure wave generated by the first pulsation of the bubble. It is also the most interesting pressure for this study, and is extracted in the final result.

The measured pressure is then compared to an analytic model of acoustic wave propagation based on the bubble dynamics derived from the Gilmore model.

## 4. Analytic Comparison

There are two major methods for modeling bubble dynamics, the direct numerical simulation (DNS) method and analytical models. The DNS method gives a more accurate result; however, it is acknowledged that obtaining DNS results is far more complex than using analytical models. Rayleigh [65] proposed the first analytical model for incompressible flow and inviscid flow. Plesset [39] accounted for viscous flow and surface tension. Gilmore [66], Keller and Miksis [68] and others accounted for compressibility effects in different ways. These analytical models cannot accurately describe the various processes of the bubble dynamics; however, they give an acceptable quantitative estimation (R(t),p(t)…) for this study with a reasonable computing time.

### 4.1. Gilmore Model

Among the analytical models presented, the Gilmore model [66] seems more appropriate for this study. Compared to Rayleigh [65] and Plesset [39] models, the Gilmore model is more accurate as it accounts the compressibility and non-linear effects, such as the sound velocity and enthalpy changes. Compared to Keller and Miksis [68] model, the Gilmore model can predict the entire process of the first growth and collapse of bubble. Details of the Gilmore model being well known, its governing equations are recalled in Appendix A.1.

### 4.2. Initial Conditions

In order to obtain the evolution of the bubble radius and pressure at the inner wall of the bubble by using the Gilmore model, two initial conditions are needed $R_{0}$ and $p_{0}$, i.e., the initial radius and initial pressure of the plasma condition. These are difficult to obtain experimentally.

For the determination of the initial radius $R_{0}$, the easiest way is to directly measure the size of the plasma spark obtained by the high-speed camera. However, compared to the camera acquisition frequency, the duration of the plasma spark is very brief (<10 ns); thus, it is not possible to acquire plasma images in every experiment. Figure 7 was selected among multiple plasma images obtained from the experiments. The maximum plasma diameter is, therefore, an estimation over a few cases.

This shows an example of a plasma snapshot luckily acquired with an estimated plasma diameter of about 120 μm. The resolution of the image does not allow an accurate value of the plasma size, and this value may be overestimated because of the local camera sensor saturation. This measured value will be taken as the maximum $R_{0}$, and an uncertainty interval will be considered with the theoretical value introduced in the next paragraph.

Another method for estimating the initial radius $R_{0}$ is to estimate the spot size of the laser beam $\omega_{0}$. The laser beam can be seen as a Gaussian beam and the minimum beam radius can be located at the beam waist. Figure 8 shows a schematic of the laser path through the lens set. For a perfectly Gaussian beam focused by a lens with focal length *f*, the focused beam waist after lens can be estimated as follows [77]:

(5) $$\omega_{0}=\frac{\lambda f}{\pi \omega },$$

where ω is the beam waist radius before the focusing lens, $\omega_{0}$ is the beam waist radius after the focusing lens, λ is the wavelength of the laser, and *f* is the focal length of the focusing lens (f=40 mm). Thus far, the laser beam is assumed to be an ideal Gaussian beam, and it is collimated before the focusing lens. The collimated beam is about $D_{\mathrm{beam}}=2\omega \approx 25\;mm$. According to Equation (5), $\omega_{0}$ should be equal to 0.54μm. In fact, the laser beam used in reality is not an ideal Gaussian beam; therefore, a dimensionless value for laser beam quality $M^{2}$ (i.e., the m-squared value) should be considered [78]:

(6) $$M^{2}=\frac{\pi \theta \omega_{0}}{\lambda },$$

where θ is the beam’s divergence half-angle. For the laser used, $M^{2}\approx 20.7\mathrm{rad}$ based on the specifications given by the laser manufacturer. $R_{0}$ can be estimated as follows:

(7) $$R_{0}=\omega_{0}=\frac{2M^{2}\lambda f}{\pi D_{\mathrm{beam}}}.$$

Finally, $\omega_{0}=11\mu m$—that is, the theoretical diameter of the beam waist is 22 μm. In practice, even if an aspherical focusing lens is used, the geometrical aberrations remain. The lens has a focal length of f=40 mm, and the diameter of the incident beam is estimated at 25 mm, which makes a low number of aperture $f/D_{\mathrm{beam}}=1.6$. This theoretical estimate is regarded as the minimum value of $R_{0}$, while the radius of the plasma obtained by the image measurement is regarded as the maximum value of $R_{0}$. A range of initial radii for the Gilmore model can, therefore, be established.

At this stage, the initial pressure $p_{0}$ remains undetermined. An iterative calculation of (Equation (A1), see Appendix A.1), based on the comparison between the calculated maximal radius and the experimental maximal radius, allows to define an estimate of $p_{0}$.

### 4.3. Pressure Fields Throughout the Liquid: Second Order Approximation

After determining the initial conditions, the evolution of the bubble radius *R* and the pressure on the inner wall of the bubble p(r=R) can be calculated. In order to compare with the experimental data, the propagation and attenuation of the pressure wave generated by the bubble collapse in the water need to be considered. Gilmore also proposed a prediction of the pressure field throughout the liquid $p\left(r\right)$ through a second order approximation where *r* is the distance to the center of the bubble. The model of the pressure field in the surrounding liquid of the bubble is described in Appendix A.2.

At this stage, the theoretical data given by the Gilmore analytical model and the experimental data can be preliminary compared in Figure 9. Time is normalized by the Rayleigh time of bubble collapse $t_{R}$ [65]:

(8) $$t_{R}=0.915R_{\max}\sqrt{\frac{\rho_{\infty }}{p_{\infty }-p_{v}}},$$

where $p_{\infty }$ is the ambient pressure, $p_{v}$ is the vapor pressure, and $\rho_{\infty }$ is the ambient water density. Figure 9a shows the evolution of the bubble radius during its expansion and collapse as a function of time. The blue crosses are obtained from the images taken by the high-speed camera. The red curve is given by the Gilmore analytical model for $R_{0}=25\mu m$. It can be seen from Figure 9a that the evolution of the bubble radius, obtained with the model is in good agreement with the experimental data until the first rebound.

Due to the viscosity of the liquid, surface tension and other non-linear factors, the bubble attenuation is much faster in the experiment than in the prediction of the Gilmore model. It is known from previous research that the first rupture of a spherical bubble produces the highest pressure peak, and thus the bubble change before and after the first collapse is the most interesting for determining the pressure generated by the bubble cavitation process.

The experimental pressure directly measured by the PVDF sensor, and the analytical pressure obtained by the wave propagation model are compared in Figure 9b. The Figure 9c enlarges the curve of the first peak of bubble collapse. It can be seen here that the curve obtained by the model is not exactly the same as that measured by PVDF; however, we are more interested in the peak value predicted and measured by model and PVDF. As shown on the Figure 9c, the peak value of the compression wave produced by the first collapse of the bubble matches the second-order approximation of the pressure field provided by the Gilmore model with a relative difference of 17%.

The sources of this difference will be discussed in detail in the next section. A tension phase is observed after the peak, which is likely due to mechanical effects caused by the viscoelastic behavior of the bonding to the backing. This fact was reported by Graham [79]. In this study, we focus on the maximum pressure (peak of the signal), the recovery phase is, therefore, assumed to have no, or a negligible effect, on the studied results.

## 5. Discussion

### 5.1. Effect of Curvature of Spherical Pressure Wave

According to the experimental results reported by Yang et al. [47], Philipp and Lauterborn [80], for γ<3, the boundary affects the spherical shape of the bubble. However, for 2<γ<3, the bubbles maintain a nearly spherical shape during the first oscillation. In the present work, only the first collapse is observed. The validity of the Gilmore model (and therefore the wave propagation model) is assumed for γ>2. The pressure wave generated by the first bubble collapse can be approximated as a spherical wave.

The pressure from the bubble wall at a distance *r* can be calculated from (Equation (A6), see Appendix A.2). The active zone of the PVDF sensor is a square with a certain area ( 1 mm^2^ in the present case). In this discussion, we assumed that $R_{max}$ is constant with a value of 0.8 mm, and only the distance between the bubble center and PVDF is adjusted.

When the bubble near the wall (standoff distance $d_{s}$ is small), the curvature of the pressure wave may be significant. A bias can appear since the spherical wave cannot propagate uniformly through the active area of the PVDF sensor for a given time. In order to estimate this bias, the propagation wave model is integrated over the active surface of the PVDF sensor. Figure 10 presents examples of theoretical disparity caught by the PVDF sensor for different $d_{s}$. The pressure information on PVDF was normalized by using the maximum pressure at the center of PVDF sensor.

It can be seen from Figure 10 that when $d_{s}$ is small $d_{s}$ = 1.6 mm—that is, the bubble is close to the solid boundary, the bias due to the curvature of the spherical wave is more obvious, which will cause a larger error in pressure. When the bubble is far away from the solid boundary, i.e., $d_{s}$ = 4 mm, the pressure wave is closer to a plane wave. In Figure 10, the error in pressure for different standoff distances $d_{s}$ is also given. The maximum error in pressure $\varepsilon_{\mathrm{cur}}$ due to the wave curvature is less than 3% for $d_{s}$ = 1.6 mm.

**Figure 11:**
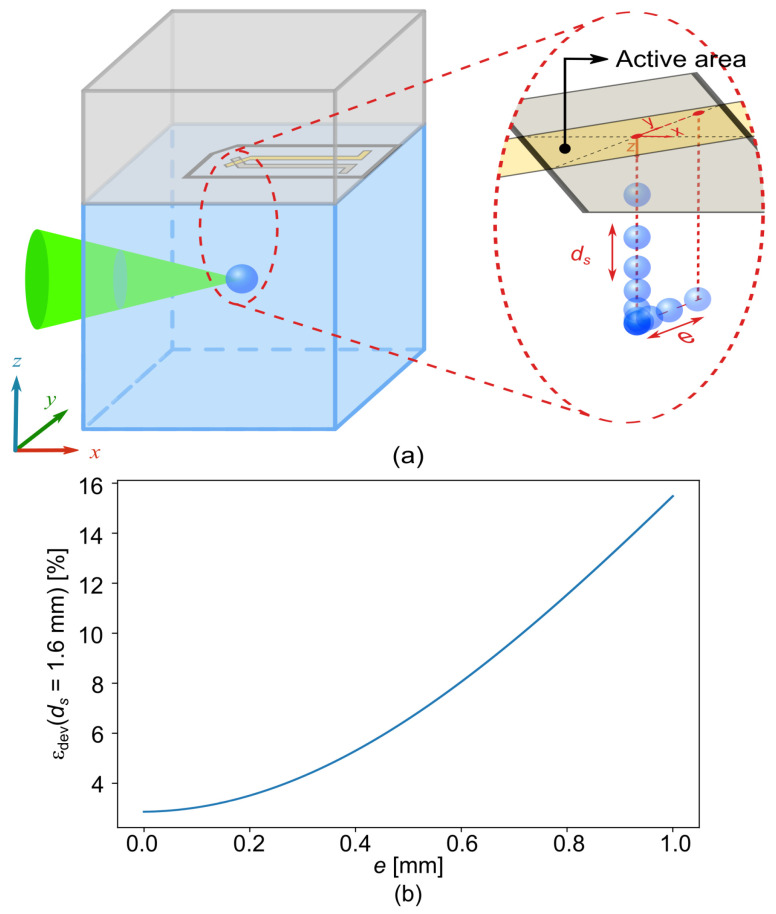
(**a**) Schematic of the bubble center and the PVDF active zone relative position. (**b**) Error in pressure due to the deviation $\varepsilon_{\mathrm{dev}}$ with a constant standoff distance $d_{s}$ = 1.6 mm for different deviation distance *e*.

An another bias called the center bias has to be taken into account. The average value of the PVDF measurement is based on ideal conditions. In particular, it is assumed that the projection of the bubble center on the PVDF sensor is perfectly located in the center of the active zone without any deviation (e=0 mm, Figure 11a). However, in practice, even if great attention is paid, this ideal situation does not exist because this alignment is difficult to obtain. Moreover, the center of the bubble is affected by the plasma position, which depends on the power of the incident laser as well as the experimental set-up. Thus, there is a certain lateral deviation between the center of the bubble and the center of the PVDF (Figure 11a).

Here, the deviation *e* is defined as the distance between the projection of the bubble center on the PVDF plane and the center of the PVDF active area with $e=\sqrt{x^{2}+y^{2}}$. Of course, $\varepsilon_{\mathrm{dev}}$ depends on the standoff distance $d_{s}$. Figure 11b shows the error in pressure due to the deviation $\varepsilon_{\mathrm{dev}}$ with a constant standoff distance $d_{s}$ = 1.6 mm for different deviation distance *e*.

A maximum misalignment of 1 mm is assumed as reasonable. This leads to a maximum error $\varepsilon_{\mathrm{dev}}$ of about 15.6% for $d_{s}$ = 1.6 mm. It is recalled that the errors $\varepsilon_{\mathrm{cur}}$ and $\varepsilon_{\mathrm{dev}}$ given for $d_{s}$ = 1.6 mm depict the most unfavorable situation.

### 5.2. Effect of Shock Wave Propagation in Different Medium

In this study, the pressure wave generated by the bubble implosion can be approximated as a plane shock wave from a certain distance (i.e., γ≳5). It is assumed that the incident shock is one-dimensional with normal incidence to the PVDF sensor. At the junction of the water surface and PVDF sensor, there is no free surface. The relaxation of the incident wave is also neglected (unloading); therefore, for the round-trip time of the shock wave propagated in the PVDF sensor, it has a sufficiently long impact time on the PVDF surface. Following Meyers [81], the conservation of the momentum applied to the medium crossed by the wave can be written as follows:

(9) $$p_{i}-p_{i-1}=\rho_{0}c\left(u_{i}-u_{i-1}\right),$$

where $\rho_{0}$ and *c* are the density and the speed of sound of the propagation medium, respectively. For each material, their values are given in Table 2.

The hydrodynamic pressure *p* and the particular speed *u* are defined by the different state of medium *i*. Generally, *i* refers to the state of upstream medium, and i−1 is the state of the downstream medium. For example, for medium water, the state 0 is the resting state (downstream) and the state 1 is the state behind the primary shock wave that propagates through the water (upstream). The acoustic impedance *Z* of a medium is defined by relation (10):

(10) $$Z=\rho_{0}c.$$

In the end, Equations (11) and (12) can be used to estimate the pressure obtained by the PVDF sensor.

(11) $$u=\frac{p_{1}+Z_{\mathrm{water}}u_{1}}{Z_{\mathrm{water}}+Z_{\mathrm{pmma}}},$$

(12) $$p=\frac{Z_{\mathrm{pmma}}\left(p_{1}+Z_{\mathrm{water}}u_{1}\right)}{Z_{\mathrm{water}}+Z_{\mathrm{pmma}}}.$$

In these equations, $p_{1}$ and $u_{1}$ are the pressure and propagation speed of the incident wave (state 1), and the pressure felt by the PVDF should quickly converge toward *p*. Therefore, it can be calculated that $p_{\mathrm{pvdf}}$ ≈130% $p_{1}$.

## 6. Result

**Figure 12:**
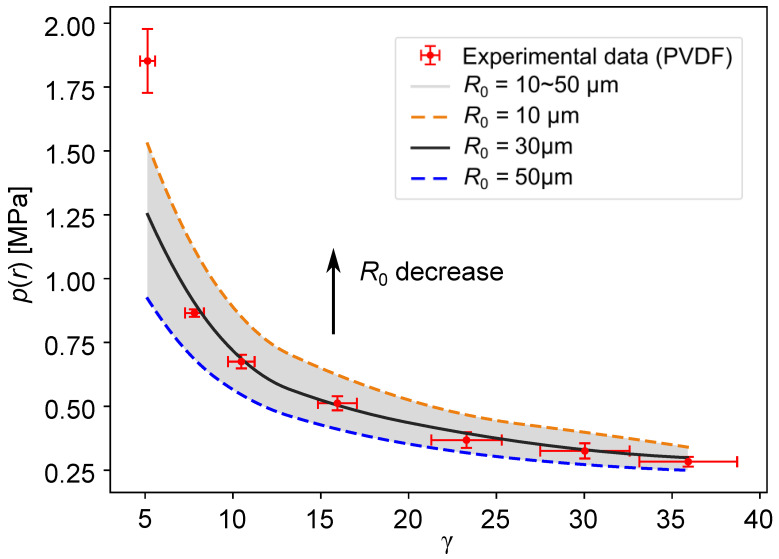
Comparison of the maximum values of the pressure wave of first collapse (red cross in Figure 5) and those predicted by the Gilmore analytical model p(r) for different $d_{s}$ with different $R_{0}$. All the measurement values contain a uncertainty estimate with a 95% confidence level.

The maximum values of the pressure wave generated by the first collapse of the bubble measured by the PVDF sensor are compared to those predicted by the Gilmore analytical model in Figure 12. The uncertainty of all the measurement values is calculated from the precision limit with a 95% confidence level of a standard deviation of the mean and the bias limit with 95% confidence estimate of the experiment equipment error. This uncertainty is represented by error bars in Figure 12. The effect of the curvature of the spherical pressure wave $\varepsilon_{\mathrm{cur}}$, $\varepsilon_{\mathrm{dev}}$ and the effect of the shock wave propagation in different mediums were superimposed on the theoretical result given by the Gilmore analytical model.

The grey area in Figure 12 represents the pressure range given by the Gilmore analytical model under different initial radii $R_{0}$ (the radius range presented in Section 4.2). The blue dotted line corresponds to an initial radius $R_{0}=50\mu m$, which gradually decreases upwards. The orange dotted line corresponds to an initial radius of $R_{0}=10\mu m$. In this area, the darkened curve is the most compatible with the experimental data i.e., $R_{0}=30\mu m$.

From Figure 12, it can clearly be seen that, when the normalized distance between the center bubble and the solid boundary is relatively large (γ>5), the maximum pressure values predicted by the model are not greatly different from those measured by the PVDF sensor. It is worth noting that, as the distance between the bubble and the solid boundary becomes smaller, that is to say, a small γ, the process of bubble collapse changes from a spherical collapse to an aspherical collapse, while the predicted pressure values given by the model still consider a spherical collapse. Thus, the results obtained by the model start to differ from the measured data.

In this study, the theoretical and measured values are in good agreement, and these results prove that PVDF sensors are a good option for quantifying a pressure wave. At the same time, it can also be inferred that, for small γ values, PVDF sensors can still quantify the pressure wave well although the measuring conditions become detrimental (micro-jet effect, lesser homogeneity of the pressure wave on the sensor’s active surface and the presence of remaining bubbles near the solid boundary after collapse).

## 7. Conclusions

Experiments of bubble collapse at various distances from a solid boundary were performed with laser-induced bubbles. The dynamics of the bubble growth and collapse were observed with rapid imaging and correlated with the Gilmore analytical model. The pressure wave generated by the bubble collapse were recorded at the solid boundary using PVDF film sensors and correlated with an analytical model of the wave’s propagation. A detailed analysis of experimental bias demonstrated that the most important source of error was due to the centering bias.

The evolution of the peak pressure at the solid boundary due to the first bubble collapse was analyzed experimentally and analytically. A very good correlation, both qualitatively and quantitatively, was obtained, especially for a normalized standoff distance greater than 5. Those results show the capability of the PVDF sensors to quantitatively measure the under water pressure waves without preliminary calibration, due to a good knowledge of the PVDF sensor properties.

## Figures and Tables

**Table 1:** Characteristics of the used PVDF sensor.

Parameters	
Stress range	10^2^ Pa to 10^10^ Pa
Frequency range	10^−2^ Hz to 10^9^ Hz
Operating temperature	−40 ${}^{\circ }C$ to 60 ${}^{\circ }C$
Piezoelectric coefficient ($d_{33}$)	23.8 ± 0.4 pCN^−1^
Thickness	0.24 ± 0.1 μm
Active surface	1 mm^2^
Input impedance of oscilloscope channels	1 MΩ

**Table 2:** The density and wave propagation speed of different materials (23 ${}^{\circ }C$).

Material	$\rho_{0}$ [kg m${}^{-3}$]	*c* [m s${}^{-1}$]
Water	998	1488
PVDF	1767	2579
PMMA	1186	2600

**Figure A1:**
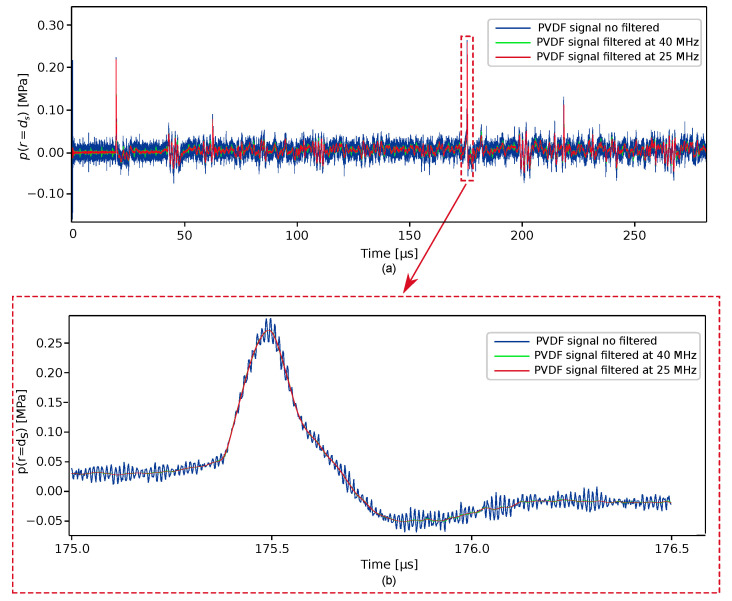
(**a**) The comparison of different filtered signals from the PVDF sensor for γ=36.4, the blue signal curve is the PVDF signal before filtering, the green curve is the PVDF signal filtered at 40 MHz and the red curve is the PVDF signal filtered at 25 MHz. (**b**) Zoom of the peak pressure caused by the bubble collapse.

## Appendix A. Formulation of the Gilmore Model

### Appendix A.1. Basic Formulas of Gilmore Model

The Gilmore model describes the motion of the bubble as follows:

(A1) $$R\ddot{R}\left(1-\frac{\dot{R}}{c}\right)+\frac{3}{2}{\dot{R}}^{2}\left(1-\frac{\dot{R}}{3c}\right)=H\left(1+\frac{\dot{R}}{c}\right)+\frac{R\dot{H}}{c}\left(1-\frac{\dot{R}}{c}\right),$$

where *R* is the bubble radius, and $\dot{R}$ and $\ddot{R}$ are the first and second derivatives with respect to time, which represent the velocity and acceleration of the bubble wall, respectively. *c* and *H* are the local speed of sound at the bubble wall and the enthalpy difference between the bubble interface and the ambient in water. Both of these parameters will change with pressure, which means that both of them will be related depending on time *t* and radius *R*:

(A2) $$c=c_{\infty }{\left(\frac{p+B}{p_{\infty }+B}\right)}^{\frac{n-1}{2n}}\;,$$

(A3) $$H=\frac{n}{n-1}\left(\frac{p_{\infty }+B}{\rho }\right)\left[{\left(\frac{p+B}{p_{\infty }+B}\right)}^{\frac{n-1}{n}}-1\right]\;,$$

where *B* and *n* are constants of the Tait state equation for liquid. From Gilmore [66], B=3040bar and n≈7 can be taken for water. $p_{\infty }$ is the environmental pressure in the water tank far from the bubble, ρ is the water density. The pressure *p* at the inner wall of the bubble is defined using Equation (A4), and the sound velocity in water $c_{\infty }$ is expressed by Equation (A5):

(A4) $$p=p_{0}{\left(\frac{R_{0}}{R}\right)}^{3k}+p_{v}-\frac{2\sigma }{R}-4\mu \frac{\dot{R}}{R}\;,$$

(A5) $$c_{\infty }=\sqrt{n\left(\frac{p_{\infty }+B}{\rho }\right)}\;,$$

Here, *k*, σ, μ and $p_{v}$ are the polytropic coefficient, surface tension, dynamic viscosity and saturated vapor pressure, respectively. All parameters used in this model are shown in Table A1.

**Table A1:** Parameters used in the Gilmore model (23 ${}^{\circ }C$).

Parameter		
Ambient pressure	$p_{\infty }$	1.0091 × 10^5^ Pa
Water density	ρ	998 kg/$m^{-3}$
Surface tension	σ	0.073N/m^−1^
Dynamic viscosity	μ	1.002 × 10^−3^ Pa/s^−1^
Vapor pressure	$p_{v}$	2340 Pa
Constant of the Tait equation	*B*	3.040 × 10^8^ Pa
Constant of the Tait equation	*n*	7
Polytropic coefficient	*k*	1.33

### Appendix A.2. Pressure Fields Throughout the Liquid

The pressure field in the surrounding liquid of the bubble is given by:

(A6) $$p\left(r\right)=p_{\infty }+\rho_{\infty }\left(\frac{j}{r}-\frac{{\dot{R}}^{2}}{2}\right)+\frac{\rho_{\infty }}{2c_{\infty }^{2}}{\left(\frac{j}{r}-\frac{{\dot{R}}^{2}}{2}\right)}^{2},$$

In the previous equation, *j* is a constant to be determined. For a given velocity $\dot{R}$ and a given pressure *p* at the bubble wall being well known, *j* can be determined using:

(A7) $$j=\frac{R{\dot{R}}^{2}}{2}+\frac{R(p-p_{\infty })}{\rho_{\infty }}\left(1-\frac{p-p_{\infty }}{2\rho_{\infty }c_{\infty }^{2}}\right).$$

## Appendix B. Comparison the PVDF Signal Filtered and No Filtered

The detailed comparison of the signal obtained by PVDF under 25 MHz, 40 MHz filter and without filter is shown in Figure A1.
